# Experience of BRCA1/2 mutation-negative young women from families with hereditary breast and ovarian cancer: a qualitative study

**DOI:** 10.1186/1897-4287-11-14

**Published:** 2013-10-16

**Authors:** Lynn Macrae, Alicia Navarro de Souza, Carmen G Loiselle, Nora Wong

**Affiliations:** 1 Department of Human Genetics, McGill University, Montreal, Quebec, Canada; 2 Department of Medical Genetics, Jewish General Hospital, Montreal, Quebec, Canada; 3 Faculty of Medicine, Federal University of Rio de Janeiro, Rio de Janeiro, Brazil; 4 Centre for Nursing Research, Jewish General Hospital, Montreal, Quebec, Canada; 5 School of Nursing, McGill University, Montreal, Quebec, Canada

**Keywords:** Cancer, Breast cancer, Hereditary cancer, Women, Non-carrier

## Abstract

**Background:**

Little is known about the experience of young women who become aware of their parent’s BRCA1 or BRCA2 (BRCA) mutation status as adolescents or young adults. There is also currently a gap in the literature pertaining to those who are found to be negative for their familial mutation. We aimed to investigate the experience of these mutation-negative young women from hereditary breast and ovarian cancer (HBOC) families.

**Methods:**

Using a semi-structured questionnaire we interviewed 8 women. All of the women were non-carriers of their familial mutation and had learned of the mutation in their family as adolescents or young adults at least 6 months prior to undergoing genetic testing. All interviews were audio recorded, transcribed, and independently analyzed by the investigators. This was followed by an in-depth cross-case analysis, enabling the formulation of emergent themes.

**Results:**

The women’s age ranged from 22 to 37 years old and all were of Ashkenazi Jewish descent. Prominent emergent themes from the interviews included the impact of how and when the familial mutation status was disclosed, the factors influencing when a young woman chooses to undergo predictive genetic testing, the predictors of post-test adjustment and risk perception, as well as the impact of familial cancer experience versus the familial mutation.

**Conclusions:**

By eliciting detailed patient narratives we have begun to show that this generation of BRCA mutation-negative young women is likely still affected by the degree of cancer history in their family, even with their understanding of the genetic contribution to disease. Larger studies with tightened participant characteristics, as well as studies involving women from different cultural backgrounds, are needed to further define the experience and needs of true negative young women from HBOC families.

## Background

Since the discovery of the BRCA genes in the mid 1990’s much information has been gained regarding the cancer risks to both men and women who carry a germline mutation in one of these two genes. The greatest risk is for women where mutation carriers have about a 50-85% risk before the age of 70 years of developing breast cancer and about a 10-45% risk of developing ovarian cancer [1]. Mutations in the BRCA genes are inherited in an autosomal dominant manner and, as a consequence, each child of a germline mutation carrier has a 50% risk of inheriting the mutation from their parent. Thus, one of the greatest concerns for individuals after learning that they carry a BRCA mutation is the risk that their children might inherit the mutation.

It is known that parents often disclose their carrier status to their adolescent children, especially mothers to their daughters [2-5]. However, there have been few studies looking at the effect that this disclosure has on children and adolescents as they mature. What has been found is that some adolescents who learn of their parent’s mutation status respond adaptively and might improve their health-related behaviour as they transition into adulthood [6]. However, little is known regarding what becomes of these adolescents as they grow to be young adults carrying the knowledge that their parent carries a BRCA mutation. Of particular interest here are the views of women growing up with the possibility of being at high risk for developing cancer and how this experience might influence the various aspects of their lives.

Given that approximately half of all women with a parent who carries a BRCA germline mutation will be negative for the gene mutation, an equally important area of research is the understanding of risk perception among non-carrier women from HBOC families. Studies have found that middle-aged women who have grown up with a strong family history of breast and/or ovarian cancer often have difficulty identifying with a population risk of cancer when they are found to be negative for their familial BRCA mutation [7]. This difficulty in accepting a lower risk is thought to be the result of a long period of cancer awareness in the family. In addition, these older women have only just come to understand the more recent possibility of the contribution of a BRCA mutation to their breast and ovarian cancer risk and might have greater difficulty incorporating this knowledge into their perception of cancer risk. It is well known that a close familial experience of breast cancer has a strong influence on a woman’s identity and how they perceive their own cancer risk [8,9]. In some cases, a genetic test might do little to erase the decades of disease experience present in the family or alter one’s cancer screening practices.

Similarly, prior to the discovery of the BRCA genes, adolescent girls developed a sense of cancer risk based on their family history and personal proximity to the disease. However, the era has now shifted where young girls might not only experience a family history of the disease, but are also growing up with the understanding of the underlying genetic cause. These young women can, in turn, decide to be tested for the underlying genetic contribution to cancer in their family. For the women who choose to be tested, those who learn they are mutation-negative might adapt differently to their test result than the middle-aged women previously described. However, given that many variables are known to be associated with risk perception [10], it is unclear if the knowledge and the understanding of genetic risk will affect identity and risk perception in the same way that family history alone has affected previous generations.

In 2003, an integrative review of the psychosocial aspects surrounding genetic testing for BRCA mutations concluded that there was a need to more systematically collect data on all those seeking genetic testing, including those who are found to be mutation-negative [11]. A decade later little has been gained with regard to understanding the experience of women who are BRCA mutation-negative. The main objective of the article is to understand the impact of being a non-carrier in young women from HBOC families. We report here on how this has shaped their lives, their risk perception prior to and post genetic testing, as well as the general effects of being a non-carrier from an HBOC family.

## Methods

### Research design

The study design was that of an exploratory cross-sectional qualitative analysis of individual interviews conducted with 8 participants ascertained through the Cancer Prevention Centre at the Jewish General Hospital in Montreal, Canada. Eligible women were those with a negative mutation status from a HBOC family, having learned of the mutation in their family before the age of 25 and at least 6 months prior undergoing predictive genetic testing. Data for each case were obtained using a modified semistructured McGill Illness Narrative Interview (MINI) [12]. The MINI model for conducting semistructured interviews is designed to elicit broad, yet detailed, patient narratives that allow researchers to examine the meaning participants give to their illness experience, as well as the impact that their experience has had on them [12]. Data obtained from each interview were analysed and coded individually followed by an in-depth cross-case analysis enabling the formulation of emergent themes. Approval for the study was obtained by the hospital institutional review board.

### Patient population

The participants were women recruited from the Department of Medical Genetics at the Jewish General Hospital. An attempt was made to identify women who were children or adolescents when their parent underwent genetic testing. We examined the pedigrees of mutation carriers who were between the ages of 25 and 40 at the time of genetic testing at least 5 years prior to recruitment for the study in 2010. This age category was chosen to increase the likelihood that their potential children would have been young at the time they were tested. We then verified if any had daughters who were tested at our institution and found not to carry their familial mutation. Eight potential participants were identified. The 8 women were contacted by phone by the principal investigator, at which time all verbally consented to an interview with the co-investigator. All 8 women subsequently participated in the interview process and their signed consent was obtained at the time of the interview. The interviews were conducted by a single investigator either at the Jewish General Hospital or at a location chosen by the patient. One interview was conducted via Skype. All participants were English-speaking, of Ashkenazi Jewish descent, and had at least one first degree relative affected with either breast or ovarian cancer. Age ranged from 22 to 37 years old (mean age 28.5 years). All had some form of postsecondary education. Four were married at the time of the interview, of whom three had two children each (Table 1).

**Table 1:** Demographic and background information

**Characteristics**	***n***
Women interviewed	8
Age at interview	
Range	22-37 years
Mean	28.5 years
Marital status	
Married	4
Single	4
Children	
Yes	3
No	5
Family history of breast/ovarian cancer	
Yes	8
First degree relative	8
No	0
Carrier parent	
Mother	7
Father	1
Communication of familial mutation	
By at least one parent	6
Self-discovered	2
Knowledge of mutation status in the family prior to testing	
Less than 1 year	2
Between 1–4 years	1
Between 5–10 years	3
Greater than 10 years	2
Religion	
Jewish	8
Christian	0
Other	0

### Data collection

Semistructured interviews were conducted using a modified version of the MINI, where “illness” was replaced with “genetic status” or “family history of cancer.” When appropriate, probing and exploration of relevant experiences shared by the participants was performed by the interviewer. As the interviews and data collection progressed, new probes and questions were formulated to elicit experiences surrounding emerging themes from previous interviews. The length of the interviews ranged from approximately 50 minutes to 1 hour and 50 minutes. The recorded interviews were subsequently transcribed verbatim for thematic content analysis and interpretation.

### Data analysis

Each transcript was transferred to a computer software program, Atlas.ti 5.2 used for coding and analysis. The complete transcript of each interview was read and coded independently by the principal investigator and the coinvestigator. Validation of the independent coding process through the random sampling of 30 passages from 2 interviews produced an agreement of 81% between the two investigators. The categories and themes produced by the coding process were derived inductively. During the coding process, codes were merged together, updated, and evolved as the data analysis progressed. After the coding was completed, thematic networks involving all transcripts were created, which allowed for the visualization and interpretation of emergent themes.

## Results

Several themes emerged from the raw data following coding and thematic analysis. A comprehensive list of emergent themes is available in Table 2.

**Table 2:** List of emergent themes

**Theme**	**Example**
Timing	There is no perfect time to undergo predictive genetic testing
	Young women appreciate learning their familial mutation status in adolescence even though concepts might not be fully understood at that time
	A balanced presentation of the genetic information for adolescent or young children is important and information needs change with time
	Having time in between learning a parent’s carrier status and undergoing personal genetic testing is appreciated, as it is used to process and contemplate the information
	Many choose to undergo predictive genetic testing at a time when they can immediately take action if found to be positive, because cancer is the problem and not the gene itself
	Age is an important determinant of when to undergo predictive genetic testing, but opinions about the best age differ
Disclosure	Young women want to learn from a parent, and prefer both parents to be present for the status disclosure of a familial BRCA mutation
	Those not informed by a parent had the desire to be tested soon after discovering their familial BRCA mutation to gain information that they had been deprived of
	Nonparental disclosure and self-discovery of a familial *BRCA* mutation might generate additional stress
Risk Perceptions	Many of the young women believe they are mutation carriers before undergoing testing
	Prototypical or familial cancer experience influences pretest and posttest cancer worry and cancer screening desires
	All understand their posttest risk of developing breast or ovarian cancer
Cancer Worry	Posttest breast cancer worry is reduced with respect to their offspring’s risk, but remains somewhat elevated for themselves
	Posttest cancer preoccupation and worry can be related to pretest levels preoccupation and worry
	Most seek an active and healthy lifestyle as a way to influence and control their cancer risk
Cancer Burden	Need for supportive counseling because of the lack of social prototype for non-carrier women
	The potential of transmitting a genetic mutation to children has an influence on decision to undergo genetic testing and potential feelings of guilt
	Weighing the pros and cons of predictive genetic testing before having children versus after having children
	Importance of the impact that genetic testing and genetic knowledge has beyond the individual
	Present and future familial and social relationships are an important factor surrounding the decision to undergo predictive genetic testing
	Feelings of survivor guilt are present in these mutation-negative young women
	The experience of hereditary breast and ovarian cancer is not over because other family members are carriers or have yet to be tested
Hope	Appreciation for the various generational differences in hereditary breast and ovarian cancer families because of the gain in knowledge and options with each successive generation
	Genetic knowledge is seen as empowering at any age
	Ability to bring awareness regarding genetic testing to their families and to others
Plans for the Future	Impact of a negative test result on the ability to plan for a future without cancer
	Preparation for a positive result with the development of a plan of action and the exploration of potential emotional reactions to this outcome
Explanatory Models for Mutation Status	Myths about non-carrier status include paternal contribution and spiritual or theological influence
	Most employ a biomedicine-based explanatory model for their carrier status

### How and when parents should disclose their mutation status

All participants believed that the genetic information in the family is something they should learn about and most expressed a desire for this information to be disclosed to them by both parents. Overall, participants recalled that limited information was provided at the initial time of disclosure and only gradually did they obtain more information from their parent or from an outside source. Participants spoke about the benefits of knowing about the mutation in the family at a young age, including having time to process the information and to gain more knowledge and understanding over time. Another benefit was the advantage of having time before the age at which decisions about screening or surgery would have to be made.

Two participants in our sample were not informed by a parent about their familial mutation. Both of these participants expressed the desire to have had both parents introduce the genetic mutation information directly to them. The experience of these individuals was that of an emotionally charged event, which is counter to what was described by the other 6 participants. They both sought genetic counseling almost immediately after discovering the information.

I was like raging mad when I found out. I mean it's hard, like I'm over it now, so it's hard to be accurate about like how I felt, but I definitely felt lied to at the time. (This 22-year-old young woman learned of the mutation in her family at 19 years old by discovering documents related to the presence of the mutation in her mother. She subsequently underwent predictive genetic testing less than one year later).

[It’s] either [you] have [the] time to deal with it. Like, process it yourself. And then just, like, take it in, then be told what it is. Or it's like: you read it, and you freak out, not know what it is, and then, it's, you're up here, and then you're like, 'Ah, okay. Oooh.’ I don't know, maybe somewhere in the middle maybe if you're having a family discussion then they bring it up. (This 26-year-old participant learned of the mutation in her family at 20 years old by discovering documents related to the presence of the mutation in her father. She subsequently underwent predictive genetic testing less than one year later).

I really appreciate the way my family handled it, being open about it even if I didn't necessarily grasp it at the age I was told. I think it's better to know and to kind of have those years to kind of ease yourself into the idea…than to be told when there's already, like, some kind of you know, sickness in the family, or newly diagnosed individual, because then it's just too much shock. (This 30-year-old woman learned that her mother is a mutation carrier in her mid-teens through an open family discussion. She underwent predictive genetic more than 10 years later).

Even when my mother did explain it to me, and, I guess, at the time, she sort of pitched it like, maybe to soften the blow, in a sense, but just that, if it did affect us, it was so many years away. Like, we were only in our twenties. (This 36-year-old woman learned of the mutation in her family in her mid-twenties through a discussion with her mother, who carries the familial mutation. She underwent predictive genetic testing more than 5 years later).

### Age is an important determinant for undergoing predictive genetic testing

Age was most often discussed with respect to the women’s decision regarding undergoing predictive genetic testing. An important age-dependent determinant for the timing of testing was the age at which the individual perceived being diagnosed with cancer to be an actual threat, that which was based on their familial or societal stereotypes of cancer. Other age-dependent factors were dating, marriage, and both career and family planning. Undergoing testing at an age that allows one time before screening and the consideration of surgical interventions was also important to those advocating testing at a younger age.

The participants were split on the issue of undergoing predictive genetic testing prior to versus after having children. The three women with children at the time of their interview all agreed that testing should wait until the completion of one’s family. Proponents of postponing testing argued that because ovaries and breasts are needed to have and to nurse children, undergoing prophylactic surgery prior to having children would not be an option, therefore genetic testing was not necessary until after. Those advocating testing before childbearing argued that the results of such testing might dictate when and even if having children would be considered.

I wouldn't wanna do [genetic testing] too late. Because what if you wanna start a family? I would say you have to do it before you start a family. Because that could play in your idea of having a family. (This 24-year-old participant learned of the mutation in her family in her late-teens and underwent predictive genetic testing at the age of 21. She did not have children at the time of testing).

Cancer is something that, you know, can affect anybody. And if you can control a little bit of it by getting tested younger, I think that people should do that. Just so that they have more options as they get older of how to minimize their risks, or to control some parts of it. (This 22-year-old woman learned of the mutation in her family in her early-teens and underwent predictive genetic testing at the age of 21. She did not have children at the time of testing).

[In] my early thirties, it wasn't as much about critical timing as if I would have been already in my forties or later forties. I finished having my kids, and after my little one was already in daycare. Because, I knew that if I did the testing, I would have made the decision to have a mastectomy pretty quickly. (This 37-year-old participant learned of the mutation in her family in her mid-twenties and underwent predictive genetic testing at the age of 34. She had two children at the time of testing).

### Posttest adjustment and risk perception

Many of the women believed they were a mutation carrier before undergoing predictive genetic testing and felt a great sense of relief after learning that they were not a carrier. However, because of the dichotomy created between their mutation-negative status and their positive familial status, conflicting emotions of relief, happiness, guilt, fear, and anger were also present. One participant even expressed a desire to have been mutation-positive.

They all understood their posttest population risk of developing breast and ovarian cancer, but some desired to have additional screening as a result of their familial cancer experience and residual cancer worry. Despite this, none were actively seeking further treatment or screening beyond what is recommended to the general population for their age category. One participant expressed a more intense ongoing residual cancer preoccupation and worry.

I have trouble believing that I'm going to escape this lifetime without getting breast cancer. And I should be thrilled that I'm negative and I am, and as every year goes on that I don't get breast cancer I know treatments are getting better and research is getting better and there is more help out there, but somehow it doesn't make me feel completely confident. (This 32-year-old woman underwent predictive genetic testing in her early thirties after knowing about the presence of the familial mutation for more than 10 years. Her mother was diagnosed with breast cancer at ages 39 and 46).

I don't feel like I need to be hyper-vigilant, or anxious about [cancer]. I don't know, I think that. I know that anxiety is not necessarily something that you can consciously control, but I did get a sense of relief from being negative and the fact I can, you know, not worry about this on a daily basis anymore. (This 30-year-old woman underwent predictive genetic testing at age 27 after knowing about the presence of the familial mutation for more than 10 years. Her sister was diagnosed with breast cancer at 29).

But I still have some chance. But at least, you know, I'm more cautious. Just cause I don't have [the mutation] doesn't mean anything. A lot of people say, 'Oh, I don't have [the mutation]. Okay, I'm okay.’ Which is not true. You know, it's just, now your chances are lower.” (This 26-year-old young woman underwent predictive genetic testing in her early-twenties, less than one year after learning about the presence of the familial mutation. Her father was diagnosed with breast cancer).

It's one of those things that you've been stressing about for so long and then afterwards it's one of those things; it's like a break-up, like you just slowly forget about it overtime. (This 22-year-old participant underwent predictive genetic testing at age 20, less than one year after learning about the presence of the familial mutation. Her mother was diagnosed with breast cancer at age 39 followed by ovarian cancer several years later).

### How the genetic knowledge has shaped their life

The general feeling among most of the women was that, even though they are non-carriers, their experience of the burden of hereditary breast and ovarian cancer is not over. They explained that their lives continued to be burdened by new cancer diagnoses in the family and the identification of other mutation-positive family members. Although this is true, many pointed to the fact that with each subsequent generation there is a gain in knowledge. This knowledge of the presence of the gene mutation in the family also shaped their lives in terms of relationships, dating, marriage, family planning, and having to contemplate one’s own mortality at a young age. Growing up with the knowledge of a genetic mutation in the family has also motivated at least several of the women to become advocates for genetic testing and for other families burdened by cancer.

I'm lucky enough not to have [the mutation], but it's a part of my family. It's still a big deal of who we are. (This 24-year-old participant underwent predictive testing at age 21 after learning of the mutation in her family in her late-teens. Her younger sister has yet to pursue predictive genetic testing).

I always thought that you know maybe if, God forbid, I have the gene I can get married a little younger, have kids, you know that way I don't need to be pressured or rushed to have kids like towards my thirties. Because if, God forbid, cancer comes around, you know, I didn't want it to be like a lingering factor always, though, like in my mind. (This 22-year-old young woman underwent predictive genetic testing at the age of 21 after learning about the mutation in her family in her early-teens. She was recently married at 22 years old).

I think it makes you think about things that you wouldn't normally think about in your twenties. I mean, I think not just dealing with the potential mortality of a parent, which is kind of normal even if it's at young age, you kind of, you know, think about that. But I don't think, at twenty, most people think about their own mortality. So, I think even in University, when everyone's just thinking about, you know, 'Where am I gonna get a job?’I was thinking about, you know, 'What will my future be?’ (This 36-year-old woman underwent predictive genetic testing at age 32 after learning in her mid-twenties that her mother, who was diagnosed with ovarian cancer, was a mutation carrier).

If it's something that you suspect is in your family, and it's available to get the genetic testing, I think people should know. You're in your fifties and then you have breast cancer, there might have been things you could have done in your forties and your thirties. I think it's always better to know, if you can, and then know what you're dealing with. So, I think that's the most important thing I try to tell people. (This 37-year-old participant learned of the mutation in her family in her mid-twenties and underwent genetic testing more than 5 years later).

## Discussion

Although each participant’s journey has been unique, there are several salient themes that can be drawn from their collective experience of being a non-carrier from a HBOC family. The more salient of which are discussed here and, if corroborated, have the potential to influence cancer genetic practice and counseling.

### How and when parents should disclose their mutation status

Many factors are known to influence the communication of genetic risk within families including the perceived relevance of the information, closeness of family members, family patterns and rules, as well as the type, amount and timing of the information being disclosed [13]. After receiving a positive BRCA genetic test result, knowing when and how to disclose this information to one’s offspring is a concern for many parents.

Learning that their parent carried a deleterious BRCA mutation as an adolescent or young adult did not seem to have any obvious negative consequences among the participants in our study. Bradbury and colleagues also report that offspring learning their parent’s carrier status before the age of 25 have a good understanding and do not express any negative consequences pertaining to the disclosure [6].

In contrast, we identified two participants who were not told of their parent’s carrier status by way of direct parental communication. Rather, both discovered documents pertaining to the presence of the mutation in their parent. This lack of disclosure is not necessarily surprising given the complexities and dilemmas faced by parents and the fact that disclosure does not occur 100% of the time [14]. The experiences of these two young women were understandably distinct from those introduced to the notion of a genetic mutation in the family by at least one parent. In particular, their reaction of seeking genetic counseling and testing soon after learning that their parent was a BRCA carrier might have been because of the need for an explanation and reassurance with regard to the crisis created by this unanticipated discovery.

### Age is an important determinant for predictive genetic testing

According to our participants, it can be said that there is no “perfect” time to undergo genetic testing, there exists only a time that is “better” than others. There are many factors that dictate when might be the most appropriate time in a woman’s life for testing.

Since the clinical availability of BRCA testing there has been much controversy with regard to testing adolescents and young adults, as is the case with many other adult-onset hereditary conditions. Although age is certainly a key factor, the ideal age to have genetic testing is personal and is perceived differently by each individual. We found that a precise age might be less important than other factors in one’s life when considering genetic testing. In fact, in her review, Pasacreta found that age was not associated with one’s interest in pursuing BRCA genetic testing and its significance in the actual utilization of genetic testing remained unclear [11].

Among our participants, timing to be tested also appeared to be influenced by the women’s general state at that moment in time. To emphasize, five of the eight women were going through a period of stress and instability in their lives, according to the Holmes and Rahe Social Readjustment Rating Scale, when they made the decision to undergo predictive genetic testing [15]. These stress-inducing life events included the unexpected discovery of the familial mutation, a new cancer diagnosis in a first degree relative, and a pregnancy. Although on the surface these might appear to be inappropriate periods for one to have genetic testing, it can be said that these women, because of the crisis and sense of instability, were ripe for change and willing to incorporate the new knowledge that genetic testing would bring.

Taken all together, age is important when considering the key psychosocial factors predicting the most appropriate time for a woman to undergo BRCA genetic testing, however it should not be considered the sole determinant for testing. It is also important to remember that the decision to know one’s mutation status is much different than the decision to act on it.

### Predictors of posttest adjustment and risk perception

One of our main objectives was to describe the adjustment and risk perception of mutation-negative women after having undergone genetic testing. Although not formally assessed with standardized items, the participants described the ways in which cancer and their mutation status has affected them in ample detail.

Posttest risk was accurately expressed by the women in our study as being similar to the population risk level, yet most had a self-professed moderate degree of cancer worry. This is not surprising given the results of various studies, such as that by Quillin and colleagues [16]. The authors found that cancer worry did not significantly correlate with risk perception in daughters of mothers with breast cancer. In addition, they found that cancer worry appeared to be stable across all risk levels, whether their actual risk of breast cancer was high or low [16]. Similarly, a longitudinal study looking at psychological distress one year following the disclosure of BRCA predictive test results found that, while the distress levels of non-carriers decreased somewhat over time, there was no significant difference in breast cancer specific distress between carriers and non-carriers [17]. It is possible that we have observed a similar trend, in that, cancer worry is elevated regardless of whether a woman from an HBOC family is at high or low risk of developing cancer. The underlying common variable between these groups of women is a family history of breast cancer. This phenomenon might help to explain the fact that the women in our study were able to accurately state their lifetime breast cancer risk, but many did not necessarily *feel* at this population risk.

Although many had a continued sense of cancer worry, none of the women were actively seeking cancer screening beyond what was recommended for the general population. However, it will be interesting to know whether these women do go on to pursue additional breast or ovarian cancer screening in the future. In fact, it has been previously shown that the cancer screening practices of mutation-negative woman from HBOC families far exceeds what is recommended. It is not clear, however, if this excess screening is patient-driven or physician-driven [18].

Furthermore, when compared to the older women studied by Bakos and colleagues [7], adjustment and risk perception of the young women in our study are similarly influenced by their personal and familial experience with cancer, even with a rational understanding of genetics. Thus, for this younger generation of genetic savvy women, coping as a mutation-negative member in a HBOC family is still proving to be complicated by the surrounding factors experienced by the generation preceding them. Therefore, what might be of interest in the future are the experiences of subsequent generations of women whose parents were among the predictive genetic testing generation. In this case, their familial experience with cancer might be very different. For instance, affected relatives might become more distanced from this future generation given that mutation carriers might remain unaffected as a result of risk-reducing procedures or because cancer might become a less devastating disease due to improved screening options and treatments. Just as was the case with the families studied by Kenen and colleagues [19], the participants in our study utilized their social and familial contexts when making sense of their risk and how they wished to manage it.

It is important to note that we are observing these women as a cross-sectional snapshot of their experience and their cancer risk perception and preoccupation might not necessarily be stable over time. At the time, only one participant expressed ongoing cancer preoccupation and worry despite the fact that her absolute risk of developing breast and ovarian cancer was clearly understood. Further evaluation her experience is necessary to build a better sense of what the underlying factors causing this persistent preoccupation might be.

Using previously reported findings, it possible to begin to formulate hypotheses with respect to this anomalous patient in our study group. For one, it is known that both general and cancer-specific distress at baseline is highly predictive of later psychological responses in members of BRCA mutation-positive families [11]. Hilgart and colleagues have also observed that reactions to one’s cancer risk assessment are greatly influenced by preconceived expectations about risk as well as one’s personal and family history of cancer [20]. Therefore, it is possible that the patient had elevated stress levels and cancer worry prior to undergoing genetic testing, which might partially explain her degree of posttest anxiety and worry. As a result of waiting more than 10 years after learning about the mutation in the family before undergoing genetic testing, this participant truly embodied the notion that she was mutation-positive. This resulted in extensive preparation for being a carrier and little time spent anticipating a negative result which, according to the participant, had contributed somewhat to her current state of maladjustment. Ultimately, a patient’s reaction to a genetic test result, whether positive or negative is not constructed in isolation, but within the context of their family history of cancer, their testing experience, and the results of their other relatives [11].

Another approach to understanding this participant is by way of heuristics, which are inferential cognitive shortcuts. Heuristics are often employed in the study cancer genetics, particularly with respect to its influence on risk perception among members of hereditary cancer families. To emphasize, Kenen and colleagues reported that women utilize three main heuristics when interpreting their breast and ovarian cancer risk, that of the representative, availability, and illusion of control [19]. Women use these heuristics to put complex or upsetting knowledge into perspective in the hopes of simplifying complicated decision making [21]. Within the narratives of our study, all three of these heuristics were repeatedly employed by the women, especially when attempting to make sense of any residual cancer worry after learning they were non-carriers.

With respect to this unique participant with excess cancer worry, the “simulation heuristic” might have more relevance in explaining her current state. The simulation heuristic was first described by Kahneman and Tversky in 1982 as a mental operation that brings things to mind by way of mentally constructing scenarios that allows one to answer complex questions by using this simulation model [22]. Normally, we would consider a negative predictive genetic test result to be a positive event and would expect reactions such a joy and a sense of relief given the alternative outcome. However, this participant anticipated being a carrier and even stated that part of her wished to have been mutation-positive for various reasons. Therefore, her view of the positive and negative outcomes of genetic testing had been reversed from the norm. Consequently, her mental simulation of being mutation-positive might have resulted in intensified emotions and feelings of regret and disappointment. This type of reaction is in keeping with studies involving the theory of the simulation heuristic [23]. In brief, in using concepts such as the simulation heuristic, we can begin to appreciate exaggerated reactions experienced by BRCA mutation-negative women. With this approach, there exists the potential to learn how to adapt genetic counseling to anticipate and reduce this type of adverse reaction.

### How the genetic knowledge has shaped their life

Without doubt, understanding how the knowledge of the presence of a BRCA mutation in a parent has impacted or shaped the lives of young women is a difficult task. It is often challenging to distinguish what has been the impact of a potential genetic predisposition to cancer versus the familial experience of cancer itself. From the narratives of our participants it is clear that the effects have been widespread and significant as a result of learning about a gene mutation that is responsible for their familial cancer experience. It can be argued, however, that some of the effects on marriage, family planning, and prophylactic surgery, for example, were caused not only by the presence of the gene, but also their unique experience with how the gene mutation has manifested itself in their family.

In some cases, the presence of the gene mutation has also enabled the young women to redefine their family history of cancer. The sense of endless suffering felt by the women can be attributed to the presence of the familial gene mutation. However, because they are non-carriers, there is a sense of freedom that has allowed them to focus on others in the family, as well as their community at large. For some, especially where prophylactic surgery was a planned and accepted reality, the fact of being a non-carrier has certainly altered the course of their life.

Ultimately, this “second generation” of women to undergo predictive BRCA genetic testing are growing up with a strong family history of cancer in addition to the genetic knowledge and their lives and identities have been shaped by both of these realities. As Margaret Lock and colleagues emphasized, it is unlikely that this genetic information will supplant one’s other identity claims. Rather, it is more likely that this genetic identity will combine and interact with other identities [24]. This is made apparent by our group of women, whose cancer experience came first, and for whom the genetic knowledge has only added a layer to their overall experience of being a young women growing up in such an environment.

To our knowledge this is the first study to report on this particular group of women in such detail. Employing a qualitative approach with our highly selected group of women provided the opportunity to explore a complex topic without the restrictions of specific hypotheses. While a sample of 8 is deemed sufficient for in-depth exploration [25], some might argue that a larger sample size might have provided more variation in the emerging themes. We later verified if any additional women would have qualified at our institution by searching our clinical databases for non-carriers. One hundred and ninety-six families were identified as carrying a BRCA mutation at our institution as of 2010. In these families, 117 women were tested and were found not to carrier their familial BRCA mutation. Of these 117 non-carriers, any women who was 40 years or older at the time of genetic testing was eliminated. The age 40 was chosen as the cut-off because the youngest they would have been at the time of disclosure would have been the mid-twenties given that the BRCA genes were discovered in the mid-1990’s. Forty two women remained. We then verified if the carrier parent was tested at least five years prior to the disclosure of their own test results. Only the 8 eligible women remained who were selected as described above. It was not possible to identify our eligible non-carriers by this method in 2010 because our clinical database linking non-carriers to persons with a mutation was not created until 2012. Therefore, this audit confirmed that our participant selection in 2010 was not bias. Using our selection process, we were unable to capture all potential non-carrier women from our carrier families because they may have been tested elsewhere, have chosen not to be tested, or have no knowledge of their parent’s BRCA mutation status. Previous research has shown that patients do disclose their genetic status to first-degree relatives but the rate of BRCA testing in at-risk relatives is relatively low [2,26]. We could have increased the number of potential participant by decreasing the time interval between the parent’s and offspring’s genetic test. However, it is unclear if this would have allowed sufficient time for the family to experience their BRCA status. In addition, by reducing the time lag, the potential non-carriers would not have been adolescents but young adults at the time of disclosure. Also, a time lag of at least 5 years did not guarantee that non-carriers were aware of their parent’s genetic status for the same interval because we did not know when disclosure occurred, as illustrated by several of our cases.

Another potential limitation is that the participants were all English-speaking and Ashkenazi Jewish. This population does represent a subset of women presenting for genetic testing at our center, but given that cultural background plays a central role in shaping one’s life, important differences might exist among other HBOC families. Another important element of homogeneity was that the women all had at least one first degree relative affected with breast or ovarian cancer. This proximity to disease and familial experience of cancer has likely significantly contributed to their overall experience as a mutation-negative woman in a mutation-positive family. We also acknowledge that the women’s age range, as well as the time since disclosure of the mutation in their family, was rather broad. As outlined earlier, because of the restrictions with regard to age and timing of genetic testing, only a limited number of women were eligible to be contacted.

## Conclusions

Testing for mutations in the BRCA genes has now been available for more than a decade and a half. During this time, research has uncovered many of the psychosocial elements surrounding BRCA genetic testing, as well as the dynamics of HBOC families. At this time we have the beginning of a new cohort of young women who have been aware of their parent’s mutation status since their adolescence and have subsequently chosen to undergo genetic testing as young adults. Prior to this study, little was known about this group of young women, particularly those who are found to be non-carriers of their familial mutation. By eliciting direct patient narratives we have found that this group of women is likely to be affected in different ways by the degree of cancer history in the family, even with their understanding of the genetic contribution to the disease.

Just as with older non-carrier women from HBOC families, this younger generation still appears to carry a heavy burden related to being a member of a HBOC family. Will this still be true of subsequent generations of HBOC families where we hope to see less cancer burden through early screening and intervention? In the meantime, the detailed narratives of these women have provided insight that has the potential, through additional research, to influence current cancer genetic practice and counseling. Larger studies with tightened participant characteristics, as well as studies involving women from different cultural backgrounds, are needed to further define the experience and needs of true negative young women from HBOC families. Of particular interest are whether residual cancer worry in these young women will translate into an unnecessary increase in cancer screening in the future and whether a better social prototype for non-carrier women will be available so that they may have the support needed to help understand their risks as well as their place within their family.

## Abbreviations

BRCA: BRCA1 and BRCA2; HBOC: Hereditary breast and ovarian cancer; MINI: McGill illness narrative interview.

## Competing interests

The authors declare no competing interests.

## Authors’ contributions

LM participated in the design of the study, conducted all interviews, provided thematic analysis of the data, and drafted the manuscript. ADN provided intellectual expertise and insight throughout the study. CGL provided critical analysis and final approval of the manuscript. NW conceived of the study, participated in the design and coordination of the study, provided thematic analysis of the data, and helped to draft the manuscript. All authors read and approved the final manuscript.

## Authors’ information

LM holds a MSc. and is a faculty lecturer in the Departments of Medicine and Human Genetics at McGill University, as well as a genetic counselor at the Jewish General Hospital, Montreal, Quebec, Canada. ANS holds a MD and PhD. and is an associate professor in the Psychiatry Department, Faculty of Medicine at Federal University of Rio de Janeiro. Rio de Janeiro, RJ, Brazil, as well as a postdoctoral fellow at the Division of Social and Transcultural Psychiatry, McGill University. Montreal, Quebec, Canada. CGL holds a RN and PhD. and is an associate professor in the School of Nursing at McGill University, holds Christine and Herschel Victor/Hope and Cope Chair in Psychosocial Oncology, and is program leader of the Psychosocial Oncology Research Training program, as well as senior Fonds de la Recherche en Santé du Québec researcher at the Jewish General Hospital. Montreal, Quebec, Canada. NW holds a MS and is a faculty lecturer in the Departments of Medicine and Human Genetics at McGill University and a genetic counselor at the Jewish General Hospital. Montreal, Quebec, Canada.
